# Realisation of magnetically and atomically abrupt half-metal/semiconductor interface: Co_2_FeSi_0.5_Al_0.5_/Ge(111)

**DOI:** 10.1038/srep37282

**Published:** 2016-11-21

**Authors:** Zlatko Nedelkoski, Balati Kuerbanjiang, Stephanie E. Glover, Ana M. Sanchez, Demie Kepaptsoglou, Arsham Ghasemi, Christopher W. Burrows, Shinya Yamada, Kohei Hamaya, Quentin M. Ramasse, Philip J. Hasnip, Thomas Hase, Gavin R. Bell, Atsufumi Hirohata, Vlado K. Lazarov

**Affiliations:** 1 Department of Physics, University of York, York YO10 5DD, UK; 2 Department of Physics, University of Warwick, Coventry CV4 7AL, UK; 3 SuperSTEM Laboratory, SciTech Daresbury Campus, Daresbury WA4 4AD, UK; 4 Center for Spintronics Research Network, Osaka University, Osaka 560-8531, Japan; 5 Department of Electronics, University of York, York YO10 5DD, UK

## Abstract

Halfmetal-semiconductor interfaces are crucial for hybrid spintronic devices. Atomically sharp interfaces with high spin polarisation are required for efficient spin injection. In this work we show that thin film of half-metallic full Heusler alloy Co_2_FeSi_0.5_Al_0.5_ with uniform thickness and *B2* ordering can form structurally abrupt interface with Ge(111). Atomic resolution energy dispersive X-ray spectroscopy reveals that there is a small outdiffusion of Ge into specific atomic planes of the Co_2_FeSi_0.5_Al_0.5_ film, limited to a very narrow 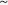
1 nm interface region. First-principles calculations show that this selective outdiffusion along the Fe-Si/Al atomic planes does not change the magnetic moment of the film up to the very interface. Polarized neutron reflectivity, x-ray reflectivity and aberration-corrected electron microscopy confirm that this interface is both magnetically and structurally abrupt. Finally, using first-principles calculations we show that this experimentally realised interface structure, terminated by Co-Ge bonds, preserves the high spin polarization at the Co_2_FeSi_0.5_Al_0.5_/Ge interface, hence can be used as a model to study spin injection from half-metals into semiconductors.

Achieving atomically controlled abrupt heterointerfaces enables engineering of electronic properties of the heterostructure such as band alignment, Schottky barrier height, interface conductivity and magnetism. Hence it is the aim of continuous experimental and theoretical research to control chemical intermixing arising due to interdiffusion, as well as interface strain, which are the main parameters that control the structural and chemical quality of a given heterostructure. However, tailoring the atomic structure of ferromagnet/semiconductor interfaces, of crucial importance for spintronic applications, has been shown to be a rather elusive goal despite the intensive research efforts over the past years^1^,^2^.

Efficient spin-injection from ferromagnets into semiconductors is essential for the development of hybrid ferromagnet-semiconductor spintronic devices such as spin transistor^3^,^4^,^5^. In addition to the film/substrate lattice match as well as thermodynamically stable interface, one of the main challenges in the field is the conductivity mismatch between ferromagnets and semiconductors, which prevents efficient spin-injection^6^. A possible solution for this problem is to use halfmetalic ferromagnets i.e. materials that are 100% spin-polarized at the Fermi-level^6^. The Co-based full Heusler alloys are an ideal candidate for such applications due to their predicted 100% spin-polarization, high magnetic moment and Curie temperature well above room temperature^3^,^7^,^8^,^9^,^10^,^11^. Co_2_FeSi_0.5_Al_0.5_ (CFAS) is a full Heusler alloy which belongs to this group of materials and in addition has a mid-gap Fermi-level, which makes it more robust against temperature effects^12^. Significant research activities have been devoted to create half-metal/semiconductor interfaces. Recent work has shown that CFAS/Si interface suffers from a number of challenges to meet the requirements for a chemically and structurally abrupt ferromagnet/semiconductor interface^2^. Due to the strong out-diffusion of Si (even at room temperature) as well as the sizable lattice mismatch of ~4.5% between CFAS and Si, non-magnetic thermodynamically stable phases (i.e. Co and Fe silicides) are formed across a large ~3 nm interface region^2^, hence ruling out this heterostructure as a technologically important candidate for spintronic applications.

In this letter, on the case of CFAS/Ge we demonstrate that atomically sharp half-metal/semiconductor interfaces are achievable with almost no strain due to the excellent lattice match between CFAS and Ge. We show that the film has the desirable *B2* ordering which provides high spin polarisation and it does not form any secondary phases in the interface region. Based on state-of-the-art aberration-corrected high angle annular dark field (HAADF) scanning transmission electron microscopy (STEM) imaging we have determined the exact atomic structure at the interface and were able to construct a realistic interface model. Density functional theory (DFT) calculations show that this interface atomic structure preserves both the high spin-polarization of the CFAS film as well as its magnetic moment in the interface vicinity, making this system an excellent platform for spin-based device applications. Finally, we show that the Ge diffusion into the CFAS film is very small. Atomic resolution energy dispersive X-ray spectroscopy (EDXS) as well as electron energy loss spectroscopy (EELS) chemical mapping reveal small and selective out-diffusion of Ge within a ~1 nm region of the interface. This atomic plane selective diffusion process does not change the structural integrity and spin-electronic structure of the CFAS since the outdiffused Ge selectively substitutes only Fe and Si/Al atoms, which in turn does not affect the film’s half-metallicity. Furthermore, by using polarized neutron reflectivity (PNR) measurements we show that the magnetic moment sharply decreases across the interface.

## Results and Discussion

**Figure 1:**
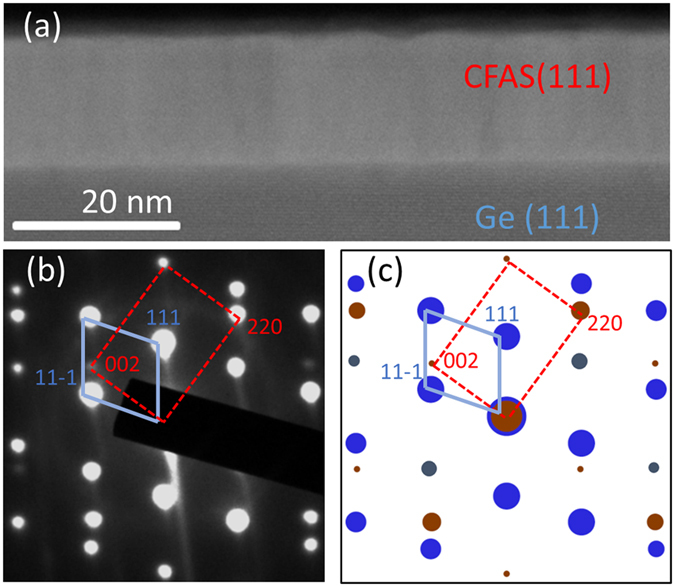
(**a**) Low magnification overview HAADF STEM image showing that the film is uniform with a thickness of ~18 nm. (**b**) SAED pattern from an area that includes both film and substrate, showing the single crystal nature of the grown film and the epitaxial relationship with the substrate. The motifs are labelled with the red dashed rectangle for the film and blue solid romb for the Ge substrate. (**c**) Simulated SAED diffraction pattern assuming twinned epitaxy with respect to the substrate which shows excellent agreement with the observations. The reflections labelled in red are from the film; in blue from the substrate. The reflections in grey appear due to double diffraction in the substrate.

First we present the overall structure of the CFAS half-metallic film grown on a Ge(111) substrate. Figure 1a is a low magnification HAADF STEM image of the CFAS/Ge(111) heterostructure. The mass contrast shows a uniformly grown CFAS film with a thickness of ~18 nm and the formation of a sharp interface with Ge. The single crystal nature of the film is demonstrated by the selected area electron diffraction (SAED) pattern recorded from an area that includes both film and substrate, Fig. 1b. Taking into account the excellent lattice parameter matching between CFAS and Ge (only 0.2% mismatch), for the case of a simple cube-on-cube epitaxy (Supplementary Figure S1) one would expect an overlap of the film’s and substrate’s diffraction reflections. However, the diffraction pattern reveals that the epitaxy with the substrate is different, i.e. there is a twinned epitaxy determined by the following epitaxial relationships: CFAS(111)||Ge(111) and CFAS(1–10)||Ge(−110). The simulated diffraction pattern shown in Fig. 1c calculated assuming the twinned epitaxial relationship shows excellent correspondence with the experimental diffraction pattern. A similar twinning phenomenon at interfaces has been also observed when a CFAS film is grown on Si(111)^2^.

Before proceeding further we briefly outline the structure of the full Heusler alloy CFAS. CFAS contains four interpenetrated fcc sublattices; two of them occupied by Co (leading to a simple cubic Co sublattice), one by Fe and one by Si/Al which share the same sublattice. This is the *L2*_*1*_ordered CFAS structure. Along the [111] crystallographic direction the *L2*_*1*_ phase consists of the following atomic planes stacking sequence …Co, Fe, Co, Si/Al, … (Supplementary Figure S2). When full intermixing of the atomic species between the Fe and Si/Al sublattices occurs, the CFAS has so called *B2* ordering. In this case each of the *L2*_*1*_ Fe and Si/Al sublattice is occupied by equal amounts of Fe and Si/Al. Besides the *B2* disorder this structural phase of CFAS is still desirable since DFT predicts unaltered magnetic moment and spin-polarization at the Fermi level^2^.

**Figure 2:**
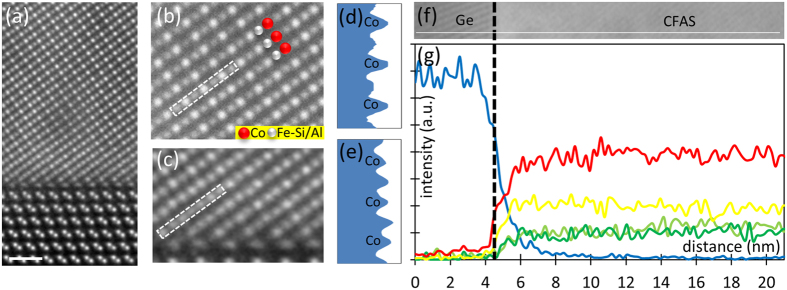
(**a**) Overview HAADF STEM image along the [1-10] crystallographic direction, showing the abruptness of the CFAS/Ge interface. The white scale-bar corresponds to 0.7 nm. (**b**) Atomic resolution HAADF STEM image from the bulk-like film region showing the distinctive Co-(Fe-Si/Al) atomic planes repeat pattern along the [001] crystallographic direction characteristic for *B2* ordered CFAS. The line profile along the white dashed rectangle is given in (**d**). (**c**) Atomic resolution HAADF STEM image from the interface region showing that the distinctive Co-(Fe-Si/Al) atomic planes repeat pattern along the [001] crystallographic direction is lost due to out-diffused Ge atoms from the substrate. The line profile along the white dashed rectangle is given in (**e**). The STEM images in (**a**,**b**) and (**c**) are obtained by rigid registration of a stack of images recorded in quick succession (resulting in high signal-to-noise and precision in the image). (**g**) Intensity signal coming from the *K*_*α*_ edge of Ge –blue curve, Co - red, Fe –yellow, Si –dark green, Al – light green; recorded during an EDXS linescan along the white solid line in (**f**).

Next we reveal the atomic structure and ordering of the deposited CFAS film by performing atomic resolution HAADF STEM imaging along the [1-10] crystallographic direction (Fig. 2a). Performing HAADF microscopy along the [1-10] crystallographic direction of the CFAS is desirable since each of the atomic species Co, Fe, Si/Al is distributed in separate atomic columns. Taking into account the ~*Z*^*2*^ dependence of the contrast in these images, where *Z* is atomic number for each element, an initial chemical identification can be performed straightforwardly. Due to the full intermixing between Fe and Si/Al in the case of a *B2*-ordered film one expects alternating high (Co planes)-low (Fe-Si/Al planes) contrast along neighbouring atomic columns. This type of ordering can be identified by following the intensity profile from HAADF images along the [001] direction. Such intensity profile is presented in Fig. 2d from which it can be easily concluded that the film has *B2* ordering^2^. The film’s *B2* ordering (Fig. 2b) extends up to the interface with the substrate except for ~1 nm region in the vicinity of the interface, Fig. 2c. The distinctive pattern of alternating Co (brighter) and Fe-Si/Al atomic columns (darker) present away from the interface is lost in this region. This is clearly seen from the intensity profile (Fig. 2e) taken along the same direction in the interface region (Fig. 2c), which reveals an increase of the intensities of the columns identified as Fe-Si/Al in the bulk-like part of the film. Such intensity increase on the atomic columns can appear if Ge atoms (brighter in HAADF STEM images) diffuse into the CFAS film, as discussed below.

In order to obtain detailed insight into the exact chemical structure across the interface we perform chemical analysis by combining atomic resolution EDXS and EELS. Fig. 2f is an overview HAADF STEM image, with an overlaid white line marking the path along which an EDXS line-scan is acquired. The intensity profiles obtained from the *K*_*α*_ edges during the EDXS linescan are presented in Fig. 2g. The most striking feature is the particularly steep decrease of the Co-signal, compared to the more gradual decrease of the Fe, Si and Al within a ~1 nm region at the interface. In addition, the Ge signal gradually decreases when approaching the film from the substrate, showing the small outdiffusion into the CFAS film over a ~1 nm region. These features of the elemental EDXS profiles support the atomic column intensity variations in the HAADF STEM image in Fig. 2c. In other words, there is Ge out-diffusion into the film substituting Fe-Si/Al atoms, which leads to the increased contrast on these atomic columns.

**Figure 3:**
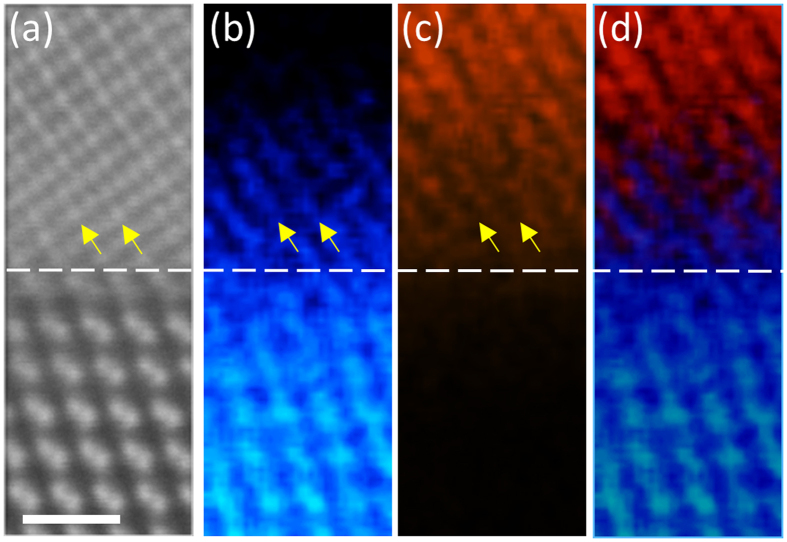
EDXS chemical mapping across the interface. (**a**) HAADF STEM image acquired simultaneously with the EDXS maps which serves as a reference image. The white scale-bar corresponds to 0.7 nm. (**b**) Ge *K*_*α*_ edge map showing the selective outdifussion of Ge across the reference interface plane (white dashed line). Yellow arrows are inserted as a guide to the eye. (**c**) Co *K*_*α*_ edge map showing the abrupt decrease of the Co signal across the interface. (**d**) Ge and Co overlaid map clearly showing the out-diffused Ge atoms in the Fe-Si/Al atomic planes which are in-between the Co(001) planes.

The spatial distribution of Ge in the interface region is further studied by atomically resolved chemical mapping performed simultaneously with the HAADF STEM imaging. Figure 3a is a HAADF STEM image in the vicinity of the CFAS/Ge interface acquired simultaneously with the EDXS maps presented in Fig. 3b,c and serves as a reference image for the EDXS maps. Fig. 3b is the EDXS map formed from the Ge *K*_*α*_ edge, while the Co *K*_*α*_ edge map is shown in Fig. 3c. From Fig. 3b the distinctive Ge ‘dumbbells’ away from the interface can be clearly seen. Above the reference interface plane (labelled with a dashed white line as a guide to the eye) a decaying Ge intensity signal is observed which confirms the conclusions made based on the lower magnification EDXS linescan, i.e. presence of Ge out-diffusion into the CFAS film over a ~1 nm region. This map shows that the out-diffusion of the Ge atoms into the film has a substitutional nature and that it is selective (as indicated by the HAADF imaging), i.e. the out-diffusion of Ge atoms is constrained along the (100) Fe-Si/Al planes. Additionally the Co *K*_*α*_ edge map shown in Fig. 3c confirms that Co remains on its original lattice i.e. the increased intensity of the Fe-Si/Al planes in the HAADF STEM images cannot be attributed to a Co-related disorder. The described phenomenon of selective diffusion is clearly summarized in the map presented as Fig. 3d which is produced by overlaying the Co and Ge elemental maps. Same localisation of the Ge and Co signals was also observed in atomically-resolved EELS maps, obtained independently and presented in Supplementary Figure S3, which strongly supports our conclusion that there is a selective diffusion of Ge into the CFAS film along specific atomic planes.

The observed phenomenon of selective atomic plane diffusion can be easily correlated with the fact that Co_2_FeGe exists as a bulk full Heusler alloy and has a lattice parameter close to Co_2_Fe(Si,Al) (~0.9% mismatch). Hence, these similarities between Co_2_FeGe and CFAS make the substitution of Si/Al by the out-diffused Ge atoms from the substrate very likely. Once Ge is incorporated in the CFAS lattice, it remains localised on Fe-Si/Al planes since the energy barrier to move on the Co sublattice is very high. We performed DFT calculations to compute the substitutional energies for Ge-Fe as well as Ge-Co swaps and the results show that swapping Ge with Fe costs 0.5 eV, while the Ge-Co swap requires energy of 2.5 eV. This very large energy difference explains why a selective diffusion is observed.

In addition, we study the influence of the out-diffused Ge on the spin-electronic structure of the CFAS. In order to address this point we performed DFT calculations on selected configurations constructed when Ge atoms substitute Fe and Si/Al in the bulk CFAS unit cell, presented in Table 1. The results show that even a large concentration of Ge into the CFAS unit cell does not affect the 100% bulk spin-polarization, while the magnetic moment is only slightly changed, as shown in the Table 1. Hence the small incorporation/doping of Ge into the film does not bring detrimental effects for the spin-electronic structure. In contrast to the case when Ge is in Fe-Si/Al columns, Ge – Co swaps would be hugely detrimental to the electronic structure; they can locally even reverse the sign of the spin-polarization^13^. However, as discussed above, the energy required for this disorder is rather large, hence this is neither expected nor observed in the chemical mapping analysis.

Next we provide insight into why the formation of secondary phases at the CFAS/Ge interface is not observed, in contrast to the CFAS/Si interface^2^. We compared the formation energies of bulk- Co_2_FeGe + bulk-Ge with the energies of Fe_3_Ge + Co_2_Ge which are representatives of stable Ge compounds with Fe and Co. In order to make the comparison straightforward we keep the number of the atoms in the compared phases constant. The energy difference between 3*Co_2_FeGe + Ge and Fe_3_Ge + 3*Co_2_Ge (secondary-phases) is 0.12 eV/atom, which clearly is not in favour of forming the secondary germanium phases. In contrary, for the case of CFAS/Si the energy difference between (3*Co_2_FeSi + 10*Si) and (Fe_3_Si + 6*CoSi_2_) is −0.23 eV/atom, showing a thermodynamic drive to form silicides at the interface between Si and CFAS, which has also been experimentally observed^2^.

The change of the structure and chemistry at interfaces can significantly affect the spin-electronic structure locally^14^; for example the interface spin-polarisation can be reduced or reversed which leads to spin-scattering phenomena detrimental to the spin-injection efficiency into the semiconducting substrate. Hence determining the spin-polarisation at interfaces is important in designing optimal systems for applications. Next by determining the exact interface atomic structure we construct a realistic interface model on which we perform DFT to compute the spin-polarisation across the CFAS/Ge interface.

**Figure 4:**
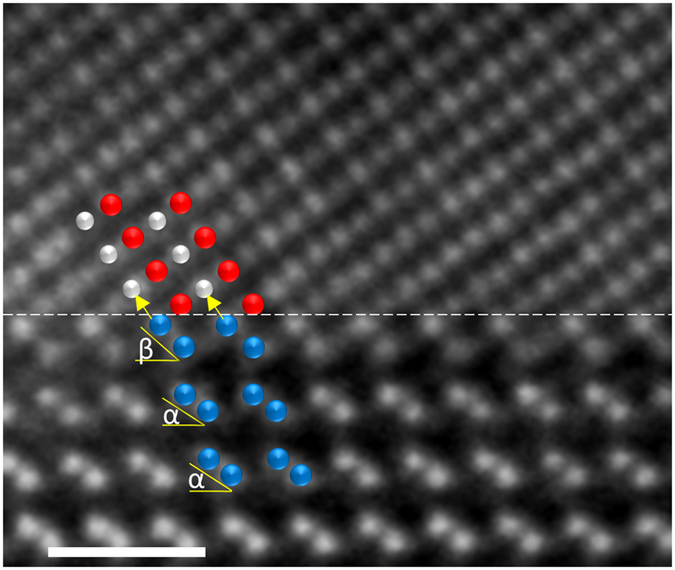
Atomic resolution HAADF STEM image of the interface showing that the CFAS film terminates on a Co(111) atomic plane. This image is obtained by rigid registration of a stack of images of the same area recorded in quick succession (resulting in high signal-to-noise and precision in the image). The colour coding of the overlaid structural model is as follows: Ge –blue; Co –red; Fe-Si/Al –grey. The white scale-bar corresponds to 0.7 nm. The tilt angle of the Ge dumbbells from α = 37° in the bulk-like region increases to β = 51° for the interfacial Ge bilayer.

Figure 4 is a HAADF STEM image of the interface, where the dashed white line indicates the interface plane clearly separating the film from the substrate. Starting from the bulk-like CFAS region (above the reference interface plane) when approaching the substrate the atomic plane stacking sequence: …-Co-(Fe-Si/Al)-Co-(Fe-Si/Al)-… characteristic for bulk *B2* ordered CFAS is observed. It can be noticed that the nearest (111) atomic plane to the substrate is a Co (111) plane. Hence, the CFAS film terminates on this plane. The bilayer below the reference interface plane is a Ge dumbbell layer. It can be observed that due to the interface bonding the dumbbells of the Ge interface bilayer are tilted by an additional (β-α) ~ 15°, as illustrated in Fig. 4. Note that the Ge dumbbells in [1-10] projection have an angle of α = 37° – with respect to the [11–2] direction i.e. interface plane. Chemical mapping has revealed the incorporation of a small amount of diffused atoms from the film into the substrate as well as Ge atoms in the film’s Fe-Si/Al atomic planes. As can be observed, this small out-diffusion of Ge does not change the Heusler-fcc structure of the film neither does the diffusion from the film change the diamond structure of Ge. Taking into account the above discussion and the finding that Ge does not affect the spin-electronic structure of the film, an interface model that neglects the small intermixing is well representative for the atomic structure of the CFAS/Ge interface.

**Figure 5:**
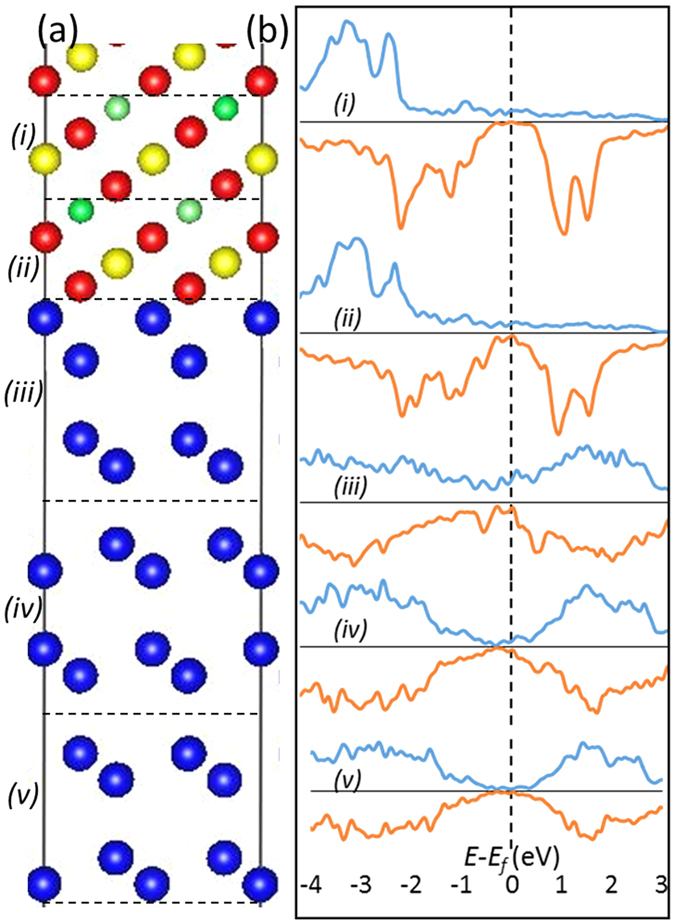
(**a**) Structural model of the CFAS/Ge interface viewed along the [1-10] crystallographic direction. (**b**) Spin-polarized PDOS for the regions labelled as (i–v) in (**a**). Spin-up PDOS are presented in the upper part of the plots, while spin-down in the lower part of the plots.

The interface model is shown in Fig. 5a after the performed DFT geometry optimization. For clarity we split the interface into five regions labelled as (*i*)-(*v*) and for each of them we plot the spin-polarized PDOS shown in Fig. 5b. It can be seen that in the regions away from the interface i.e. layer (*i*) and layer (*v*) the bulk-like features of the CFAS and Ge, respectively, are recovered. The interfacial CFAS region (*ii*) shows that near the Fermi-level there is a small number of spin-down interfacial states; however this does not significantly affect the spin-polarization at the Fermi-level which is still very high. Similarly, due to the interface bonding a small number of states emerge into layer (*iii*); yet this layer is significantly positively spin-polarized. In other words, the sharp interface preserves the very high spin polarisation of the CFAS in the interface vicinity, a property that is highly desirable for spintronic applications. Finally, we show that the sharp atomic interface model reproduces the distances and angles of the interfacial Ge dumbbell bilayer with respect to the interfacial Co layer from the CFAS film. Figure 5a shows the relaxed coordinates of the atomic layers in the interface region. As in the experiment, the interface Ge bilayer shows the same tilting angle in comparison to the angle in the bulk Ge dumbbells, hence confirming that the effect of the small interdiffusion on the structural interface property is negligible. In other words, the Co-Ge bonding is dominant and ensures the structural and electronic integrity of the CFAS/Ge interface.

**Figure 6:**
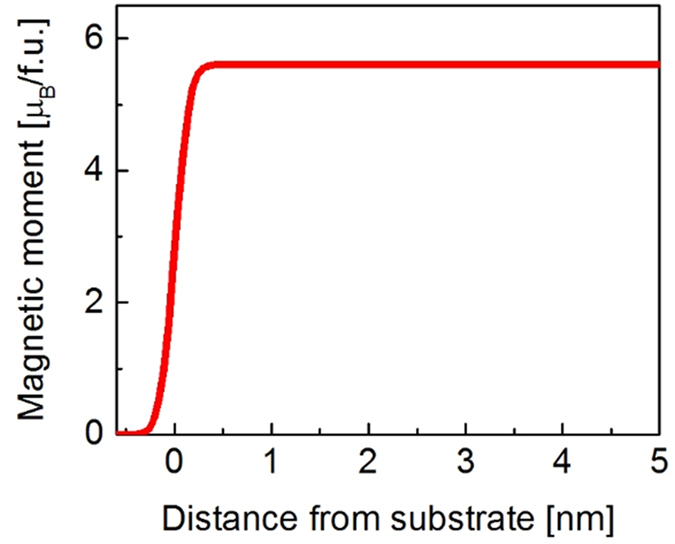
Magnetic profile across the CFAS/Ge interface obtained by PNR measurements. Value zero on the distance axis corresponds to the interface.

These conclusions were further confirmed by polarized neutron reflectivity (PNR) measurements which enable the determination of the magnetic profile of the CFAS film across the interface. By fitting the scattering length densities (SLD) for the neutrons and x-rays, both structural and magnetic information on the macro-scale for this heterostructure were obtained (Supplementary Figure S4). Figure 6 shows that the magnetic moment abruptly decreases from the CFAS bulk-value to zero in the substrate, over two atomic planes (~0.5 nm). These experimental results are fully consistent with the DFT predictions and EDXS/EELS observations that Ge out-diffuses on the Fe-Si/Al planes, hence affecting neither the magnetic moment nor the spin-electronic structure, as expected for an atomically sharp interface.

In summary, we showed that a Co_2_FeAl_0.5_Si_0.5_ film deposited on Ge(111) substrate has *B2* ordering and forms an abrupt interface with the substrate. Atomic resolution imaging and spectroscopy performed by HAADF STEM and EDXS/EELS revealed that the out-diffusion of Ge, limited to a very narrow 1 nm interface region, is of a substitutional nature and that it is selective to only Fe-Si/Al atomic planes. By employing aberration-corrected electron microscopy we revealed the exact atomic structure at the interface which was shown to be determined by Co-Ge bonding. Density functional theory calculations showed that this particular bonding preserves the high spin polarization of the Heusler film across the interface. Polarized neutron reflectivity measurements confirmed the chemical and atomic study results by showing an abrupt decrease of magnetic moment at the interface from the bulk CFAS value to zero in the substrate. This study demonstrates that half-metallic Heuslers can form atomically and magnetically sharp interfaces with Ge that preserve high interfacial spin-polarization. Hence this heterostructure provides a model of ferromagnet/semiconductor system highly desirable for spintronic applications as well as fundamental spin injection studies.

## Methods

The samples were prepared by co-deposition of Co, Fe, Si and Al using low-temperature molecular beam epitaxy^15^,^16^. 18 nm-thick CFAS film was deposited on a pre-cleaned 10 × 10 mm^2^ Ge (111) substrate at room temperature. Prior to loading Ge(111) substrates into the chamber, their surfaces were chemically cleaned with an aqueous 1% HF solution to remove any native oxide and contamination.

Cross-sectional transmission electron microscopy samples were prepared by Focused-Ion-Beam (FIB). SAED patterns were recorded using a JEOL-2011 TEM microscope. Simulated diffraction patterns were produced using the CrystalKit software package. Atomic-level structural studies were performed by HAADF STEM imaging on a Nion UltraSTEM 100 microscope, operated at 100 kV, with a convergence angle of 30 mrad; at these optical conditions the electron probe size is determined to be 0.9 Å; the inner detector angle for HAADF STEM imaging and the EELS collection angle were 76 mrad and 31 mrad, respectively. The native energy spread of the electron beam for the EELS measurements was 0.3 eV; with the spectrometer dispersion set at 0.2 eV/channel, this yielded an effective energy resolution of 0.6 eV. EELS chemical maps were generated from spectrum images after denoising by Principle Component Analysis, using the CiMe plugin^17^ for Digital Micrograph by integrating the signal above the relevant ionisation edge onset over a 30 eV window, after subtraction of the decaying background using a power-law model. Further atomic-level STEM analysis was performed using ARM200F microscope with probe and image aberration CEOS correctors operating at 200 kV. Annular Dark Field images were obtained with a JEOL annular field detector with an inner angle of 70 mrad; fine imaging probe of ~23 pA and convergence semi-angle of ~22 mrad. EDXS analysis was performed with probe currents of approximately 200 pA and collected with a windowless Oxford Instruments X-Max Silicon Drift Detector with area of 100 mm^2^.

DFT calculations were performed with the CASTEP^18^ code using a periodically repeating supercell which contains two equivalent interfaces. The supercell is large enough (28 Ge and 13 CFAS atomic planes along the [111] direction) so that the CFAS and Ge bulk-like electronic structure is recovered in the regions away from the interfaces. The PBE+U exchange-correlation functional^19^ was used, where the Hubbard-U term was set to 2.1 eV for both d-block elements Co and Fe^20^. This value for the Hubbard-U term has previously been shown to open up the minority band-gap, approximately correcting for the delocalising effect of self-interaction with PBE alone^21^. The plane wave cut-off energy was set to 600 eV, while the Brillouin zone was sampled using a Monkhorst-Pack grid with a *k*-point sampling spacing of 0.03 2π Å^−1^. The atomic coordinates as well as lattice parameters were fully geometry optimized. The partial density of states (PDOS) were calculated with the OPTADOS code^22^ using the fixed Gaussian broadening scheme.

PNR measurements were recorded at room temperature using a wavelength range of 1–15 Å in time of flight (TOF) mode on the POLREF reflectometer at ISIS, Rutherford Appleton Laboratory, UK. A saturating 1.5 T external magnetic field was applied during the measurement. The data was fitted simultaneously with X-ray reflectivity (XRR) using the GenX software package^23^. The XRR data was recorded with a Panalytical X’pert Pro MRD using λ_Cu(*Kα*)_ = 1.54 Å.

### Data Availability

All data created during this research are available by request from the University of York Data Catalogue https://dx.doi.org/10.15124/e3f87d05-ab3c-49ef-a69d-0a9805b77d2f.

## Additional Information

**How to cite this article**: Nedelkoski, Z. *et al*. Realisation of magnetically and atomically abrupt half-metal/semiconductor interface: Co_2_FeSi_0.5_Al_0.5_/Ge(111). *Sci. Rep.* **6**, 37282; doi: 10.1038/srep37282 (2016).

**Publisher’s note**: Springer Nature remains neutral with regard to jurisdictional claims in published maps and institutional affiliations.

## Supplementary Material

Supplementary Information

## Figures and Tables

**Table 1:** Magnetic moment (μ_B_/unit cell) and spin-polarization values (%) of configurations *c1-c8* obtained when Ge atoms gradually substitute the Fe-Al/Si atoms in the bulk CFAS unit cell.

label	n (Fe)	n (Si/Al)	n (Ge)	magnetic moment	spin-polarization
CFAS bulk	4	4	0	22.0	100
c1	4	3	1	22.0	100
c2	4	2	2	23.0	100
c3	4	1	3	24.0	100
c4	4	0	4	24.0	100
c5	3	0	5	20.0	100
c6	2	0	6	15.8	69
c7	1	0	7	11.2	88
c8	0	0	8	7.0	24

‘n’ stands for the number of atoms per unit cell for each of the atomic species.
