# Supplementary material for: Realisation of magnetically and atomically abrupt half-metal/semiconductor interface: Co2FeSi0.5Al0.5/Ge(111)
Source: Sci Rep. 2016 Nov 21;6:37282. doi: 10.1038/srep37282 (PMC5116668; doi:10.1038/srep37282)
Supplement: Supplementary Information [file srep37282-s1.docx]

**Supplementary Information for:**

**Realisation of magnetically and atomically abrupt half-metal/semiconductor interface: Co_2_FeSi_0.5_Al_0.5_/Ge(111)**

Zlatko Nedelkoski^1^, Balati Kuerbanjiang^1^, Stephanie E. Glover^2^, Ana M. Sanchez^2^, Demie Kepaptsoglou^3^, Arsham Ghasemi^1^, Christopher W. Burrows^2^, Shinya Yamada^4^, Kohei Hamaya^4^, Quentin M. Ramasse^3^, Philip J. Hasnip^1^, Thomas Hase^2^, Gavin R. Bell^2^, Atsufumi Hirohata^5^ and Vlado K. Lazarov^1*^

^1^Department of Physics, University of York, York YO10 5DD, UK

^2^Department of Physics, University of Warwick, Coventry CV4 7AL, UK

^3^SuperSTEM Laboratory, SciTech Daresbury Campus, Daresbury WA4 4AD, UK

^4^Department of Systems Innovation, Osaka University, Osaka 560-8531, Japan

^5^Department of Electronics, University of York, York YO10 5DD, UK

***email: vlado.lazarov@york.ac.uk**

**
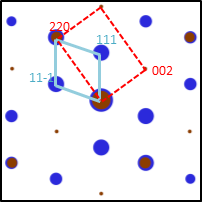
**

**Supplementary Figure S1.** Simulated SAED diffraction pattern assuming simple cube-one-cube epitaxy with respect to the substrate. This pattern shows the overlap of the film’s and substrate’s reflections as expected, due to the excellent lattice match. Colour coding is the same as in Figure 1.


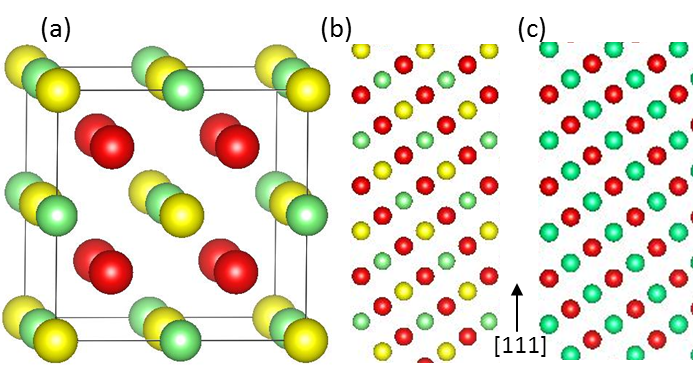


**Supplementary Figure S2.** Structural model of *L2_1_* ordered CFAS viewed along a) arbitrary crystallographic direction b) along the [1-10] crystallographic direction. c) *B2* ordered CFAS along the [1-10] crystallographic direction. Colour coding is as follows: Co –red; Fe – yellow, Si/Al –green, Fe-Si/Al – grey.

**
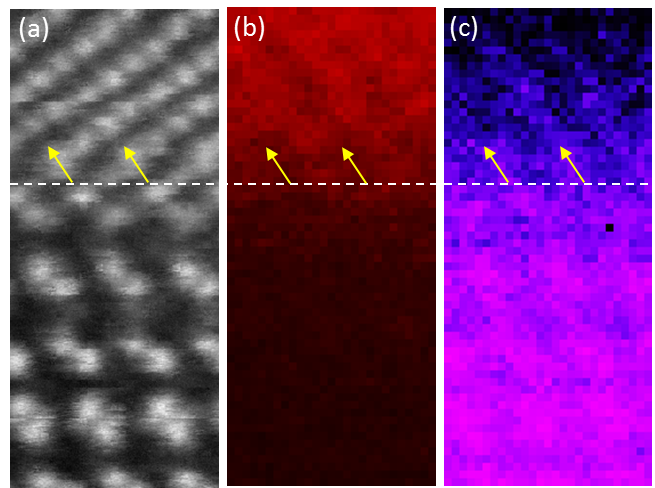
**

**Supplementary Figure S3.** EELS chemical mapping across the interface. a) HAADF STEM image acquired simultaneously with the EELS maps. b) Co *L_2,3_* edge map showing the abrupt decrease of the Co signal across the interface. c) Ge *L_2,3_* edge map showing the selective out-diffusion of Ge across the reference interface plane (white dashed line). Yellow arrows are inserted as a guide to the eye.


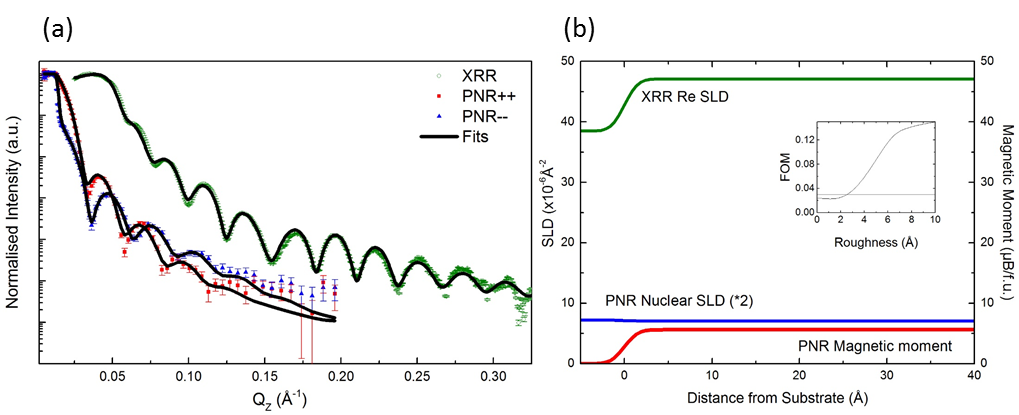


**Supplementary Figure S4.** a) Simultaneous fit of XRR and PNR data, where R++ and R-- denote neutron parallel and antiparallel polarizations with respect to the external magnetic field. b) Scattering length densities (SLD) for the neutrons and x-rays as a function of distance from the interface; PNR profile shows the sharp decrease of the magnetic moment at the interface and from XRR an interface roughness of ~ 1.2 Å is inferred. The inset in (b) shows a parameter scan of the substrate roughness in terms of the figure of merit (FOM). The roughness of 1.2 Å is the value for the best fit with lowest FOM value of 0.02298. The CFAS film has magnetic moment of 5.6 ± 0.3 µ_B_/(formula unit) which agrees very well with the theoretically predicted value for bulk CFAS.
